# Supplementary material for: Antecedents of loneliness among cancer survivors: An exploratory analysis of the Health Information National Trends Survey (HINTS) data
Source: PLoS One. 2026 Jan 30;21(1):e0323159. doi: 10.1371/journal.pone.0323159 (PMC12857977; doi:10.1371/journal.pone.0323159)
Supplement: Attachment — Submitted filename: Response to reviewers.docx [file pone.0323159.s002.docx]

**Reviewer #1:**

 Thank you for the chance to review the paper. The only noticeable flaw is the limited number of patients in the urban setting. Other limitations have been stated in the limitations section. This paper can inspire many future researchers to investagate the topic further.

**Response: Thank you very much. We have acknowledged it as a limitation.**

**Reviewer #2:**

Thank you for the opportunity to review this manuscript, which explore antecedents of social isolation and their impact on mental health among cancer survivors. This is a strong and well-executed paper. I recommend only minor revisions to further strengthen its conceptual clarity and interpretive depth.

One central question concerns the relationship between social isolation and loneliness (SIL) and mental health. A complicating factor is the likely bidirectional relationship between these constructs. SIL may contribute to the development of mental health challenges, but it may also arise as a consequence of such conditions—for example, anhedonia and social withdrawal are hallmark symptoms of depression. I encourage the authors to briefly address this bidirectionality in the discussion section, even if only to note the limitations of causal inference in the current design.

**Response: Thank you for this important observation. We agree that the relationship between social isolation and loneliness (SIL) and mental health is likely bidirectional, with each influencing the other over time. In the revised Discussion section, we have acknowledged this complexity by noting that while SIL can increase vulnerability to mental health challenges such as depression and anxiety, these same conditions can, in turn, exacerbate SIL through mechanisms like social withdrawal, reduced motivation, and anhedonia. We have also highlighted the need for future longitudinal or causal modeling studies to disentangle these temporal dynamics and better understand how interventions might interrupt this feedback loop.** 
A similar consideration applies to variables related to negative perceptions of life, which may serve both as predisposing risk factors and as symptoms of depression Acknowledging this complexity would strengthen the interpretive nuance of the paper.

**Response: We have acknowledged this as well.**

The application of theory and statistical modeling is logical, rigorous, and clearly explained. The incorporation of the Stress Process Model is a strength. As described, this model highlights the effects of cumulative stress over time. However, it was unclear to what extent the cumulative nature of stress exposure was captured in the analytic model. If not explicitly modeled, the authors may consider discussing how the model could inform future work or how the cumulative nature of stress might shape the interpretation of their findings.

**Response:** Y**ou are correct in noting that the cumulative nature of stress exposure, as emphasized in the SPM, was not directly captured in our statistical model due to the cross-sectional nature of the data. Our use of the SPM was primarily to provide a theoretical foundation for understanding the interrelated psychosocial and health constructs examined in the study. We agree that modeling cumulative stress over time would offer important additional insights, and we have acknowledged this limitation in the manuscript. We have also noted in the future research section that longitudinal designs are needed to explore how stress accumulates and evolves, and to examine its dynamic effects on outcomes such as social isolation, mental health, and life perception. We have revised the manuscript to further clarify this point and ensure readers are aware of the theoretical versus empirical application of the model in this context.**

**Reviewer #3:**

The study addresses a relevant topic, focusing on a determinant of mental and physical health: social isolation in cancer survivors. It uses a nationally representative database (HINTS 6), allowing important generalizations to the US population. PLS-SEM modeling is suitable for examining complex and mediating relationships in contexts with multiple latent variables. The division between urban and rural areas introduces an important contextual dimension to the analysis, thereby expanding the understanding of geographic disparities in cancer care.

Overall, this is a rigorous and pertinent study that contributes to the understanding of the factors associated with social isolation and its relationship with mental health among cancer survivors. However, the manuscript could benefit from some improvements, mainly in the detailing of certain information (such as the sample), in the discussion of the implications of the limitations in the results, and in including a section on the practical implications of the obtained results.
1. A more detailed description of the sample is missing, mainly in terms of sociodemographic and clinical variables. Although the study includes basic information, the authors should include a more complete table of sociodemographic and clinical characteristics (such as age, ethnicity/race, income, type of cancer, etc.). It may make sense to separate the descriptive statistics between urban and rural areas (since this is a focus of the study). This more detailed description provides greater richness to the study and contextualization.

**Response: We agree. We have added a new table with participant characteristics.**

2. Since this is a cross-sectional study, limiting causal inferences about the relationships proposed in the theoretical model, it would be pertinent to include: a more explicit discussion about the limitations of causality in cross-sectional studies; suggest future longitudinal studies to validate the proposed relationships; reflect on possible reversibility bias in the observed relationships (e.g., isolation → poorer perceived quality of care).

**Response: We agree and have revised the future work section as per your suggestion. We did previously acknowledge the causality limitation but in this revision, we have added the potential of bi-directionality between variable as you and other reviewers identified.**

**Just to clarify, while developing the model, we did test all possible permutation and combinations of path-directions and finalized this model due to it best performance and better alignment with some existing theories.**

3. Explicitly discuss which important variables were missing in the HINTS data and how this may affect the results

**Response: We mentioned the missing data handling in the method section. “Missing values were handled using the pairwise deletion method, allowing the retention of as much data as possible while excluding only the missing values for specific analyses.”**

**PLS-SEM is designed to handle smaller data size and pairwise deletion method minimizes skewness in the results. To further safeguard the model, we used 10,000 bootstrapping iterations. Stating the same in the discussion will be redundant.**

4. All variables were self-reported; therefore, other types of bias, such as recall bias and social desirability, should be discussed.

**Response: We have this in the limitation section.**

5. Provide better support for interpretations based on existing literature on the differences between rural and urban health systems or contexts.

**Response: This is beyond the scope. HINTS don’t provide us with health systems settings. We cannot control for it. A respondent can be in a rural area but is not necessarily suffering poverty. (We don’t have this information). Additionally, our model does not account for any health system components or SEIPS model. So, comparing based on health system will only be a stretched inference not based on the direct findings. However, we did compare those for which we noted significant differences based in page 28 – 30.**

6. An essential component that is missing from the manuscript is directly suggesting practical implications based on the results obtained (e.g., public policies, contextual interventions, etc.)

**Response: We cannot suggest these based on non-causal findings. It will be scientifically incorrect.**
