# Supplementary material for: Antecedents of loneliness among cancer survivors: An exploratory analysis of the Health Information National Trends Survey (HINTS) data
Source: PLoS One. 2026 Jan 30;21(1):e0323159. doi: 10.1371/journal.pone.0323159 (PMC12857977; doi:10.1371/journal.pone.0323159)
Supplement: Attachment — Submitted filename: Response_to_reviewers_auresp_2.docx [file pone.0323159.s003.docx]

**Reviewer #1:** (No Response)

**Reviewer #2:** The authors have sufficiently address my feedback and I am happy to recommend this paper for publication.

*Response: Thank you*

**Reviewer #3:** The manuscript has been significantly improved, but still requires some minor adjustments that will further strengthen the study's contribution to the literature on cancer survivor health. The authors considered and responded to all comments and suggestions. A new table with participant characteristics was added, increasing the transparency and contextualization of the study. The discussion of the limitations of cross-sectional studies was adequately addressed. The inclusion of the potential bidirectionality between variables (e.g., social isolation ↔ mental health) and the suggestion of future longitudinal studies are strengths of the new version of the article.

*Response: Thank you for the acknowledgement*

Regarding missing variables in HINTS, the authors refer to the treatment of missing data. However, what was suggested was a critical discussion of the important variables missing from HINTS and how this may limit the interpretation of the results (examples of critical missing variables include cancer type, treatment type, access to mental health services, and support network). We suggest that the authors engage in this critical reflection.

*Response: We have now added a critical discussion of the important missing variables from HINTS in the discussion section of the manuscript.*

*“In addition to the measurement constraints, several critically important variables were absent from HINTS, further limiting the interpretability of our findings. Notably, the dataset does not capture cancer stage, treatment modality, or the strength of patients’ social and support networks, factors known to strongly influence psychological distress, coping behaviors, and health communication patterns. Other missing factors that warrant consideration in future studies include cancer recurrence status, treatment side-effect burden, functional limitations, insurance adequacy, transportation barriers, and neighborhood-level environmental exposures. These factors can shape perceived distress, help-seeking behavior, and disparities in health outcomes but could not be evaluated within the constraints of the HINTS dataset. The absence of these variables constrains our ability to contextualize the observed associations and may mask important subgroup differences or pathways. Future research should aim to validate this framework with expanded measures that include clinically, psychosocially, and structurally salient variables, ideally using datasets that incorporate these determinants and allow for longitudinal assessment.”*

Regarding the literature on rural and urban contexts, the answer that this is "out of scope" is insufficient. Statistical control for health system variables was not requested; however, theoretical foundations from the existing literature were used to interpret the differences observed between rural and urban areas. It is suggested that studies that discuss access barriers, community cohesion, or disparities in post-cancer mental health be cited to better support interpretations, even if indirectly.

*Response: We have added 8 new references. Please see ref# 29,30,35,36,37,38,40, and 41.*

Regarding practical implications, caution must be exercised and causal prescriptions avoided. However, even in exploratory studies, it is possible and desirable to suggest practical implications and plausible interventions based on the results, always with some caution. For example, telemedicine programs, virtual support groups, or health literacy initiatives could be mentioned as potential strategies, especially in rural communities. We recommend including an "Implications for Practice" subsection with possible, data-based recommendations.

*Response: We agree. We have removed and rephrased all causal prescriptions. Implications are embedded throughout the discussion. We understand that data derived recommendations are invaluable, but in the context of our study, doing will be mostly speculation and not data driven. As noted by you, the cross-sectional data allows us only this much.*
